# A Narrative Review of Factors Historically Influencing Telehealth Use across Six Medical Specialties in the United States

**DOI:** 10.3390/ijerph18094995

**Published:** 2021-05-08

**Authors:** Pavani Rangachari, Swapandeep S. Mushiana, Krista Herbert

**Affiliations:** 1 Department of Interdisciplinary Health Sciences Augusta University, Augusta, GA 30912, USA; 2 Department of Family Medicine, Augusta University, Augusta, GA 30912, USA; 3 School of Nursing and Health Professions, University of San Francisco, San Francisco, CA 94117, USA; ssmushiana@usfca.edu; 4 Department of Clinical Psychology, Rowan University, Glassboro, NJ 08028, USA; herbertk9@rowan.edu

**Keywords:** telehealth use, telehealth sustainability, telemedicine policy, medical specialties, hospital organizations, specialty societies, patient-centered care, provider culture

## Abstract

Prior to the COVID-19 pandemic, studies in the US have identified wide variations in telehealth use across medical specialties. This is an intriguing problem, because the US has historically lacked a standardized set of telehealth coverage and reimbursement policies, which has posed a barrier to telehealth use across all specialties. Although all medical specialties in the US have been affected by these *macro* (policy-level) barriers, some specialties have been able to integrate telehealth use into mainstream practice, while others are just gaining momentum with telehealth during COVID-19. Although the temporary removal of policy (coverage) restrictions during the pandemic has accelerated telehealth use, uncertainties remain regarding future telehealth sustainability. Since *macro* (policy-level) factors by themselves do not serve to explain the variation in telehealth use across specialties, it would be important to examine *meso* (organizational-level) and *micro* (individual-level) factors historically influencing telehealth use across specialties, to understand underlying reasons for variation and identify implications for widespread sustainability. This paper draws upon the existing literature to develop a conceptual framework on *macro-meso-micro* factors influencing telehealth use within a medical specialty. The framework is then used to guide a narrative review of the telehealth literature across six medical specialties, including three specialties with lower telehealth use (allergy-immunology, family medicine, gastroenterology) and three with higher telehealth use (psychiatry, cardiology, radiology) in the US, in order to synthesize themes and gain insights into barriers and facilitators to telehealth use. In doing so, this review addresses a gap in the literature and provides a foundation for future research. Importantly, it helps to identify implications for ensuring widespread sustainability of telehealth use in the post-pandemic future.

## 1. Introduction

Telehealth refers to the use of electronic media to support a broad range of remote services, such as patient care, education, and monitoring [1]. Proponents of telehealth have argued that it has the potential to transform healthcare delivery by improving access, care coordination, efficiency, reducing costs, improving patient experience, provider satisfaction, and the overall quality of care [1,2].

Prior to the COVID-19 pandemic, although telehealth was a topic of much debate and conversation in the United States (US), the use of telehealth services was restricted to select medical specialties [1,3,4,5,6]. For example, a 2018 US-based weighted survey study on the use of any form of telehealth by individual physicians in their practice (including interactive audio/video, store-and-forward telemedicine, and remote patient monitoring), identified wide variations in telehealth use by medical specialty, with allergy-immunology reporting the lowest use at 6.1%, and radiology reporting the highest use at 39.5%. In other words, only 6.1% of individual physicians in allergy-immunology reported using any form of telehealth in their practice. The following other specialties were identified as having lower telehealth use: general surgery (9.7%), gastroenterology (7.9%), obstetrics/gynecology (9.3%), and family medicine (11.8%). By comparison, the following other specialties were identified as having higher telehealth use: cardiology (24.1%), psychiatry (27.8%), emergency medicine (22.3%) and pathology (23%) [3]. Although earlier studies in the US have paid attention to the wide variation in telehealth use by specialty, the aforementioned study was the first to quantify the variation across medical specialties at the individual provider level. The results of this landmark 2018 study have already been widely referenced in the general telehealth literature, and the evidence has been corroborated in the growing ‘specialty-level’ telehealth literature during the pandemic [7,8,9,10,11,12].

### 1.1. Problem of Interest

The wide variation in telehealth use across medical specialties in the US, is an interesting and important problem to examine, since the nation has historically lacked a consistent set of policies for telehealth coverage and reimbursement, which in turn has served as a barrier to telehealth use across all specialties [13,14]. Although all specialties in the US have been affected by these *macro* (policy-level) barriers to telehealth use, as discussed earlier, some specialties have been able to integrate telehealth use into mainstream practice, while others are just gaining momentum with telehealth during the COVID-19 pandemic. Although the temporary removal of policy (coverage) restrictions during the pandemic has accelerated telehealth use across all specialties, the future sustainability of telehealth remains uncertain (for example, with respect to provider acceptance, patient preferences, and policy-level support) [15,16]. Importantly, however, there is consensus in the telehealth literature that the permanent removal of *macro* policy-level barriers by itself, would not suffice to ensure sustainable telehealth use across all medical specialties [2,17,18]. On the other hand, the current literature emphasizes the need for healthcare organizations and providers to undertake concerted, dedicated initiatives towards implementing telehealth services for sustainable use [1,18,19,20,21,22].

Calling upon the *macro-meso-micro* framework, three levels of factors, including, *macro* (societal or policy-level)*, meso* (group or organizational-level), and *micro* (individual-level) factors can help to explain behavior, e.g., telehealth use within a medical specialty [23]. Based on the above discussion, if *macro* (policy-level) factors by themselves do not help to explain the historical wide variation in telehealth use across specialties, then it would be important to examine the *meso* (organizational-level) and *micro* (individual-level) factors (barriers or facilitators) influencing telehealth use across medical specialties to gain insights into underlying reasons for the variation in telehealth use across specialties. Such insights, in turn, could provide a foundation for identifying implications to ensure the widespread, sustainable use of telehealth services in a post-COVID-19 era.

### 1.2. Purpose, Scope, and Research Questions

Over the past two decades, a considerable amount of attention has been paid to factors (barriers or facilitators) influencing telehealth use in general, at policy, organizational, and individual levels [24,25,26,27,28,29]. However, there is limited literature on factors influencing telehealth use at the specialty level, both within and (especially) across medical specialties, which in turn is essential for understanding underlying reasons for wide variations in telehealth use across specialties. This paper seeks to address this gap. The paper draws upon the existing literature to develop a conceptual framework on *macro-meso-micro* factors (barriers or facilitators) historically influencing telehealth use within a medical specialty. The framework is then used to guide a narrative review and synthesis of the telehealth literature across six medical specialties, including three specialties with lower and three with higher telehealth use in the US. The aim of the review is to address the following research questions:

1. What *macro*- (policy), *meso*- (organizational), and *micro*-level (individual) factors (barriers or facilitators) historically influenced telehealth use in six medical specialties, including three specialties identified as having lower telehealth use (allergy-immunology, family medicine, gastroenterology) and three identified as having higher telehealth use (psychiatry, cardiology, radiology) in the US?
2. Which factors (barriers or facilitators) are associated with relatively lower use of telehealth services in some medical specialties and relatively higher use in other specialties in the US?

By addressing the above research questions, this paper seeks to identify implications for ensuring widespread future sustainability of telehealth services across medical specialties. It would be relevant to note that the scope of the review is restricted to the US, because *macro* (policy-level) factors impacting telehealth use in medical specialties in the US, including coverage and reimbursement for telehealth, are unique to the US health system and, therefore, are not comparable across nations.

## 2. Existing Literature on Telehealth Use

The existing telehealth literature has consistently emphasized the importance of recognizing the complexity in implementing telehealth services for successful and sustainable use [2,29]. By definition, telehealth services are delivered over a distance and often span multiple organizational entities with varying cultures, practices, and business models. There are also multiple interdependent dimensions of telehealth to consider, including processes, user-experience, and sustainability. Correspondingly, the design and implementation of telehealth services often involves engagement of stakeholders from a variety of disciplines from both inner and outer settings of the organization, including healthcare providers, managers, administrators, patients, information and communication technologists, economists, and policy makers [2,29]. In view of this complexity, a considerable portion of the telehealth literature has paid attention to determinants of failure or success of telehealth initiatives, including factors (barriers or facilitators) influencing telehealth use and implementation [24,25,26,27,28,29,30,31,32,33,34,35,36,37,38,39,40,41,42,43,44,45].

In 2005, Yellowlees defined seven core principles for success with telehealth implementation: (1) telehealth applications should be selected pragmatically rather than philosophically, (2) clinician drivers and telehealth users must own the systems, (3) telehealthcare management and support should be from the bottom up rather than top down, (4) the technology should be user-friendly, (5) telehealthcare users must be well-trained and supported, (6) telehealthcare applications should be evaluated in a clinically appropriate and user-friendly manner, and (7) information about the development of telehealth must be shared [24]. This simple yet influential set of principles touches upon key organizational (*meso*) and individual-level (*micro*) factors influencing telehealth use, including organizational leadership, change management, technological, and individual provider level factors.

Within the last decade, van Dyk (2014) conducted a comprehensive review to identify and compare existing frameworks on telehealth use and implementation, to identify common themes and areas for future development [29]. A total of nine frameworks related to telehealth use and implementation were reviewed, including: (1) barriers to the diffusion of telemedicine, which emphasize technical, behavioral, economic, and organizational barriers; (2) telehealth readiness assessment tools, which emphasize core (planning), technological, learning, societal, and policy readiness; (3) telehealth applications of the Unified Theory of Acceptance and Use of Technology (UTAUT), which describe the interaction among several variables influencing technology acceptance, including the perceived importance of standardization; (4) the seven core principles for the successful implementation of telemedicine (discussed earlier); (5) lessons in telemedicine service innovation, which identify factors contributing to telehealth success, including the policy context, evidence gathering, outcomes monitoring, perceived benefit, reconfiguring services, professional roles, and willingness to cross boundaries; (6) a framework for assessing health system challenges to scaling up for telehealth, which includes consideration for policy, organizational, technological, and financial challenges; (7) a comprehensive model for evaluation of telemedicine, which considers several issues related to telehealth implementation, including cost of education, quality of clinical services, and community access to services, among others; (8) a layered telemedicine implementation model, which identifies determinants of success associated with each lifecycle phase of telemedicine; and (9) the Khoja–Durrani–Scott (KDS) Evaluation Framework, which also considers telehealth lifecycle stages and incorporates various themes of evaluation, including readiness and change, policy, technological, behavioral, economic, and ethical. Overall, the review by van Dyk (2014) concluded that a holistic approach is needed to telehealth implementation, which includes consideration for organizational structures, change management, technology, economic feasibility, societal impacts, perceptions, user-friendliness, evidence and evaluation, and policy and legislation [24,29,30,31,32,33,34,35,36,37,38,39]. 

In more recent years, the Consolidated Framework for Implementation Research (CFIR) has been leveraged to guide telehealth service implementation initiatives [40,41,42,43]. Since its introduction in 2009, the CFIR has gained considerable popularity and recognition as an influential theoretical framework to inform both ‘implementation science’ and ‘implementation strategy’ [40]. The CFIR comprises five major domains: (1) Intervention characteristics, (2) Outer setting (3) Inner setting (4) Characteristics of individuals and (5) Process. Each domain, in turn, is mapped to an array of constructs informed by existing implementation theories and conceptual models. For example, the domain of inner setting is mapped to the following constructs: structural characteristics, networks and communication, culture (including norms and values of the organization), and implementation climate or the absorptive capacity for change. The five domains (and constructs) in the CFIR in turn interact in rich and complex ways to influence implementation effectiveness. The CFIR is a pragmatic meta-theoretical framework with a comprehensive taxonomy, which could be used to guide formative evaluation of implementation, including the identification of potential barriers and facilitators from the perspective of the individuals and organizations involved in the implementation [40]. 

## 3. Developing a Conceptual Framework

Taken together, the substantial existing literature on telehealth use and implementation frameworks, helps to identify a comprehensive set of *macro-meso-micro* level factors (barriers or facilitators) influencing telehealth use to guide the narrative review of the literature across six medical specialties. To begin with, at the *macro* level, the frameworks on telehealth readiness assessment, lessons in telemedicine service innovation, framework for assessing health system challenges, the KDS framework, and the CFIR all point to the importance of consideration for policy-level factors, legal-ethical factors, and other societal-level structural factors (e.g., growing healthcare costs and anticipated workforce shortages) influencing telehealth use. Likewise, at the *meso* level, emphasis on the perceived importance in the UTAUT and the perceived benefit in lessons from telemedicine innovation points to the importance of considering the role of the historical perceived importance or rationale for telehealth use as a factor influencing telehealth use within the specialty. Similarly, the emphasis on organizational leadership and change management in the seven core principles, the KDS framework, and the CFIR highlight the importance of considering the influence of both hospital/health system organizational factors and specialty professional-society organizational factors on telehealth use within the medical specialty. It would be relevant to note that financial factors influencing telehealth use would be subsumed within health system organizational factors, since economic feasibility and impact consideration of telehealth investments are expected to arise at a provider organizational level, rather than at a specialty level. Next, the domain of intervention characteristics in the CFIR and the emphasis on technology across all frameworks calls for the consideration of the influence of technological factors [24,30,40]. Similarly, the emphasis on reconfiguring services from lessons in telemedicine innovation, and quality of clinical services in the comprehensive model for telemedicine evaluation, call for the consideration of the influence of treatment factors on telehealth use within the specialty. Likewise, the emphasis on evidence gathering and outcomes monitoring in lessons on telemedicine service innovation, and on intervention characteristics in the CFIR, calls for the consideration of the influence of research factors, and the emphasis on organizational culture in the CFIR calls for the consideration of the influence of cultural factors on telehealth use in the medical specialty [40]. 

**Figure 1:**
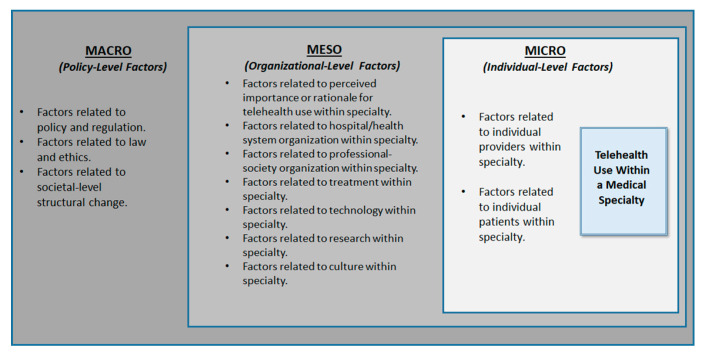
Conceptual Framework.

At the *micro* level, the emphasis on the individual (clinicians and users) in the seven core principles, Unified Theory of Acceptance and Use, and the CFIR, points to the importance of considering individual provider-level factors and patient-level factors influencing telehealth use [24,30,32,33,35,37,39,40]. In summary, the review of existing literature on factors influencing telehealth use and implementation helped to identify a total of 12 factors across all 3 levels, including 3 at the *macro* level, 7 at the *meso* level and 2 at the *micro* level. The final framework used to guide the review is summarized in Figure 1.

## 4. Narrative Review Methodology

The *macro-meso-micro* conceptual framework (summarized in Figure 1), was used to guide a narrative review of the specialty-level telehealth literature in the US to examine factors (barriers or facilitators) influencing telehealth use across six medical specialties, including three specialties with lower telehealth use (allergy-immunology, family medicine, gastroenterology) and three specialties with higher telehealth use (psychiatry, cardiology, radiology).

### 4.1. Synthesis of Themes to Generate Insights

To address the two research questions, the focus of the narrative review was to apply the conceptual framework to the specialty-level telehealth literature, to synthesize themes in regard to the *macro-meso-micro*-level factors examined, and to gain insights into whether each of the factors historically served as a barrier or as a facilitator to telehealth use within the medical specialty. For example, with respect to ‘specialty-society organizational factors’, the objective was to understand if the respective specialty societies (in the six medical specialties) historically had limited involvement (barrier) or extensive involvement (facilitator) in promoting telehealth use within the specialty, e.g., through leadership and change management to increase tech-training for providers and/or advocating for the incorporation of telehealth training in the medical residency curriculum. In other words, the review sought to apply the conceptual framework and synthesize themes with respect to each factor to gain insight into whether the factor served as a barrier or as a facilitator to telehealth use within the medical specialty.

### 4.2. Expectation of Theme Saturation

Given the focused nature of the narrative review, the expectation was that for each factor, there would be a point in the review beyond which a saturation of themes would occur, resulting in a duplication of insights (i.e., no new insights beyond the saturation point). For example, when examining the historical (pre-pandemic) influence of ‘specialty-society organizational factors’ on telehealth use in allergy-immunology, if the review indicates that the Allergy society did not release an official position statement on telemedicine until a few years prior to the COVID-19 pandemic, that the statement acknowledged the low adoption of telemedicine within the specialty, and that these findings in turn were corroborated by other articles within the specialty during the pandemic, then this would indicate a theme of ‘limited involvement by specialty society’ (barrier), and confirm the insight that the Allergy society historically played a limited role in promoting telehealth use within the specialty. Such an insight could be gained through a review of 10 or less articles meeting the eligibility criteria for review within the specialty. In other words, due to the saturation of themes, no further new insights could be gained on this factor for this specialty, neither by reviewing additional articles within the same database, nor by exploring more than one database for additional articles on the topic. For this reason, i.e., the expectation of theme saturation owing to the focused nature of the narrative review, a choice was made to restrict the article search to a single database. PubMed was the logical choice for a single database search, given that the review topic pertained to telemedicine and medical specialties. Additionally, PubMed had a total of nearly 8500 available records related to telehealth or telemedicine across the six specialties of interest, which was deemed to be more than sufficient to fulfill the aims of this narrative review.

### 4.3. Database Search

As indicated above, the article search for this narrative review was conducted on the PubMed database. The following key search terms were used “<Specialty Name>,” “Telehealth,” “Telemedicine,” “Barrier,” and “Facilitator.” Table 1 outlines the full search strategy, including the search terms and search results.

### 4.4. Eligibility Criteria

Eligibility criteria for article selection were determined based on the review’s purpose, scope, and research questions (described in the Introduction). To begin with, there were no date restrictions for the PubMed search, since the objective was to understand factors historically influencing telehealth use across medical specialties. Additionally, there were no restrictions by article type. Given the broad nature of the research questions, all forms of peer-reviewed literature (e.g., reviews, original studies, and specialty professional-society position statements) that helped to understand factors influencing telehealth use across the six specialties were included in the review. On the other hand, since the scope of the review was restricted to the US, articles that did not originate in the US were excluded. Articles were also excluded if they were not relevant to addressing the research questions. Lastly, articles were excluded if they duplicated the insights that had already been gained with respect to factors influencing telehealth use within a given medical specialty.

### 4.5. Processes Used to Explore and Synthesize Themes in the Data

To address the two research questions, the *macro-meso-micro* framework was used to guide the exploration of themes among articles within and across specialties. Within each specialty, articles were reviewed for information across all 12 factors within the framework. This process enabled the summarization of article text for each factor within the framework within each specialty, which in turn enabled a synthesis of themes within and across specialties.

The text-summaries of articles were maintained in a Microsoft Excel^©^ workbook. Each author maintained a separate workbook. All three authors independently reviewed included articles (in all six specialties) for final synthesis. Each individual workbook included six worksheets dedicated to each specialty of interest. Every worksheet was organized by layer and factor. Each worksheet in turn, contained a text-summary for all 12 factors across *macro-meso-micro* layers. The robustness of synthesis was assessed in three stages. In Stage 1, all three authors independently reviewed all articles in each of the six specialties to develop textual summaries for each factor in each specialty worksheet. In Stage 2, the three authors met several times to discuss their respective textual summaries within each specialty, to enable identification of overlapping and distinct themes across the three authors’ summaries. During the course of these discussions, all overlapping themes were acknowledged, and distinct themes were verified through a re-review of relevant articles. This process was repeated for all text summaries in the six worksheets. In Stage 3, the first author developed an integrated workbook to include the worksheets from all three authors for every specialty. The first worksheet for every specialty was updated to include the number of overlapping and distinct themes relevant to each textual summary, for each author pair. The data generated and analyzed for the study, i.e., the text-summaries of articles reviewed by factor across the six medical specialties, is included in the Supplementary Materials S1.

## 5. Results

As indicated in Table 1, the initial database search resulted in a total of 1421 articles. After removal of duplicates and non-US based studies, the total was reduced to 437 articles. Next, articles not relevant to addressing either research question were excluded (i.e., articles that did not address any of the factors examined, articles that addressed barriers or facilitators in a different context from telehealth use, and articles that were too narrow in scope were excluded). This brought the total down to 136 articles for full-text review. Following the full-text review, articles that duplicated the insights already gained were excluded, leaving a final total of 53 articles for inclusion in the review across the six medical specialties [46,47,48,49,50,51,52,53,54,55,56,57,58,59,60,61,62,63,64,65,66,67,68,69,70,71,72,73,74,75,76,77,78,79,80,81,82,83,84,85,86,87,88,89,90,91,92,93,94,95,96,97,98]. Table 2 (below) summarizes the key themes and insights that emerged from the narrative review and synthesis related to factors influencing telehealth use. These themes are discussed in greater detail in the remaining subsections within the Results section. Table 3 (provided at the end of the article), summarizes key characteristics of the 53 individual articles reviewed.

### 5.1. The “MACRO” Level

#### 5.1.1. Factors Related to Policy and Regulation

In the US, reimbursement and coverage for telehealth services are not regulated at a national level, which in turn has served as a barrier to telehealth use across all specialties [46,47]. Medicare has historically only covered telemedicine services that involved use of interactive real-time video and audio, with limited payment for store-and-forward and remote monitoring modalities. Historically, telehealth regulations also varied considerably across states and private payers [46]. Although Medicare coverage for telehealth has increased during the pandemic, there is still no standardized set of telehealth policies in the US [46,47]. It would be relevant to note that, although Medicare payments for teleradiology and telepsychiatry services have historically been more consistent relative to other specialties, teleradiology (which hardly differs from regular radiology owing to lack of in-person patient interaction) has historically faced a number of billing-related challenges from Medicare, as well as challenges related to contracting and credentialing with hospitals and healthcare organizations [92]. Likewise, telepsychiatry (which is different from other specialties since it does not require a physical exam), has faced several challenges with respect to receiving Medicare reimbursement at the level of an in-person encounter [82]. 

#### 5.1.2. Factors Related to Law and Ethics

Similar to inconsistent policies for telehealth coverage and reimbursement in US, there is considerable variation across states and payers in regard to regulations for provider licensure, credentialing, and privileging [49]. Historically, physician licensing mandates have required physicians to carry a medical license in the state of patient residence. For example, in psychiatry, each state has its own licensing boards that establish practice jurisdictions for providers licensed in the state, and some have specific regulations related to telepsychiatry. Similarly, in radiology, the regulatory and legal environment for teleradiology in the US is a limiting factor. For full/comprehensive services with final reading, radiologists need to be licensed in the remote institution’s state, credentialed in the institution, and insured for medico-legal liability. Additionally, other legal/ethical factors, such as HIPAA-related concerns, ethical issues pertaining to the privacy and security of data, and concerns associated with malpractice and cyber liability have historically served as a barrier to telehealth use across all six specialties.

#### 5.1.3. Factors Related to Societal-Level Structural Change

Telehealth use across all six medical specialties have also been influenced by structural changes at the societal level. For example, escalating healthcare costs in gastroenterology have given rise to specialty patient-centered medical homes (PCMHs) for the value-based treatment of inflammatory bowel disorders (IBDs), while other conditions in this specialty (e.g., non-infectious colitis), continue to receive traditional care [54,60]. Similarly, concerns related to projected workforce shortages in allergy-immunology are gradually influencing the specialty to favor telehealth adoption [49]. Likewise, the field of family medicine faces increasing telehealth service demand from patients, low rates of use in general primary care, and growing pressures to provide proactive population-based healthcare within a fragmented healthcare system [66,70]. In psychiatry, there is an urgent need for integrating technology into new models of mental healthcare as the demand for mental health services is soon expected to exceed the supply of providers [86,90]. In cardiology, increasing patient acceptance of wearable devices has resulted in a surge in remote monitoring of cardiac patients [73,80,81]. Likewise, billing and contractual challenges as well as the growing need for subspecialty expertise (e.g., teleradiology in pediatrics) have influenced the course of telemedicine in radiology [92].

### 5.2. The “MESO” Level

#### 5.2.1. Factors Related to Perceived Importance or Rationale for Telehealth Use within the Specialty

Among specialties with lower telehealth use, the historical rationale for using telemedicine in allergy-immunology, was to ‘improve access to care’ for underserved populations [46,47,49]. In other words, in this specialty, telehealth was not perceived as having the potential to (1) ‘improve patient experience’ (e.g., through patient empowerment for asthma control), (2) ‘reduce healthcare costs; (e.g., by decreasing hospitalizations for asthma), or (3) ‘promote population health’ (e.g., by making self-management of asthma more effective). In family medicine, physicians in primary care private practices have been found to be significantly less likely to use telehealth compared to counterparts in health system-owned practices with integrated electronic health record (EHR) systems [64,65,66]. Additionally, physicians who used telehealth were also more likely to be located in a rural setting, conveying a rationale for historically using telemedicine to ‘improve access to care’ [64]. The overall lower telehealth use in family medicine is explained by the large number of physicians who continue to provide general primary care in private practice. Similarly, in gastroenterology, although telehealth has been leveraged for the treatment of IBD (through use of specialty-PCMHs to improve quality and reduce costs), telehealth use for non-IBD conditions remains restricted to improving access to care [55,59,60].

Among the specialties with higher telehealth use, historically, telemedicine has been leveraged by providers and hospitals in cardiology to improve patient experience and reduce costs (e.g., through reduction in heart failure readmissions) [72]. Additionally, as a result of growing patient acceptance of wearable technology, remote monitoring of cardiac patients has also grown substantially [73]. In psychiatry, although telemedicine began to be used as a tool for increasing access to mental healthcare, several pioneering providers began using telemedicine to improve the patient experience [82,83,84]. In radiology, telemedicine has been historically leveraged most to meet the need for after-hours hospital-based emergent radiology coverage [92]. Patient satisfaction has also provided an incentive to use teleradiology for expediting services. Therefore, the rationale for teleradiology emanated from the combined need to preserve revenues for the profession and improve the quality of services [92].

#### 5.2.2. Factors Related to Hospital and Health System Organization within the Specialty

Historically, due to the lack of reimbursement, telehealth initiatives in the US had to be undertaken at the hospital or provider level. Such investments, in turn, were viewed as learning experiments requiring a risk taking and entrepreneurial mindset on the part of providers. It was essential to take a long-term perspective to assess the return on investment in these cases, which was often achieved through reduction in hospitalizations or optimization of in-person encounters [82]. Within this context, among specialties with lower telehealth use, hospitals and health systems have historically provided limited support for telehealth use in allergy-immunology [46,49]. On the other hand, hospitals organizations have been able to develop a ‘business case’ for telehealth use in other specialties, e.g., using telepsychiatry to grow revenues by attracting younger patients, using telecardiology to reduce costs, and earning ‘pay-for-quality’ incentives [72,78]. In family medicine, physicians based in large health systems with an integrated EHR system were more likely to use telemedicine [64]. Likewise, in gastroenterology, physicians affiliated with large integrated health systems have leveraged telehealth for IBD care [56].

Among specialties with higher telehealth use, in cardiology, hospitals and health systems had a dual incentive to use telemedicine for quality improvement and cost savings [72]. Similarly, telemedicine in psychiatry has benefitted from extensive support from hospitals, including the Veterans Health Administration, the largest health system in the US [85,86,87,88]. Likewise, telemedicine in radiology has historically received substantial support from hospitals and health systems [92,93].

#### 5.2.3. Factors Related to Professional-Society Organization within the Specialty

Among specialties with lower telehealth use, the American Academy of Allergy, Asthma, and Immunology (AAAAI) has historically had limited involvement in providing guidance on telehealth use in allergy-immunology. As recently as 2017 (three years preceding the pandemic), the AAAAI issued an official position statement on telehealth. The statement clearly acknowledged that allergy providers have historically found it challenging to get started with telemedicine because of lack of reimbursement, complexity in launching a telemedicine program, and concerns related to changing the physician-patient relationship [49]. The society has also called for hospital support of patient education, implying that historically, it has not played a leadership role in engaging patients in telehealth use [49]. On the other hand, although the American Academy of Family Physicians (AAFP) has historically played an active role in advocating for technology use in family medicine, its emphasis has largely been on policies related to meaningful use of EHRs, including EHR interoperability and the need for integrating social determinants of health into primary care to promote population health and fulfill the premise of PCMHs [68,69]. As such, before the pandemic, the AAFP paid relatively little attention to telehealth per se in primary care. Nevertheless, following the devastating impact of COVID-19 on primary care, the field has acknowledged that many barriers were present to an effective pandemic response in primary care, including an inadequate infrastructure for telehealth, clinician communication, and home hospital care. In the midst of the pandemic, field leaders have issued recommendations for redesigning primary care by adopting proactive population care through the combined use of disease registries and telehealth [66]. In gastroenterology, although the American College of Gastroenterology (ACG) has helped to support the adoption of telehealth and the adoption for IBD care within a specialty-PCMH delivery model, and it has remained more reactive in supporting telehealth adoption for non-IBD conditions within the specialty [59,60].

Among specialties with higher telehealth use, the American Heart Association (AHA) and American College of Cardiology (ACC) and have played an active role in advocating for more consistent payment policies from public and private payers to promote telehealth use in cardiology. These specialty organizations have also played an active role in helping providers get started with telehealth and overcome reimbursement challenges. Importantly, they have been proactive in educating providers on designing and implementing a sustainable telehealth infrastructure [75,76,79]. Similarly, the American Psychiatric Association (APA) has played a sustained proactive role in developing and guidelines and best practices for telemedicine in psychiatry from early stages of adoption [84]. Likewise, the American College of Radiology (ACR) has played an active role in the institutionalization of teleradiology [92,93].

#### 5.2.4. Factors Related to Treatment within the Specialty

Among specialties with lower telehealth use, research in the field of allergy-immunology has shown that a variety of treatments could be provided using telehealth, including home-based videos for triage, telehealth for antibiotic allergy, and remote monitoring for asthma management [46,47,52]. Historically however, the field has defaulted to in-clinic encounter-based care for asthma and underleveraged the unique opportunities for telemedicine in the specialty. In family medicine, a large number of providers are still engaged in the provision of general primary care in small-to-mid-size private practices, as opposed to PCMH arrangements, which are known to be more conducive to using telehealth.64 In gastroenterology, telehealth is being increasingly used to treat and coordinate care for individuals with IBD, while it is utilized less for non-IBD conditions [55,57].

Among specialties with higher telehealth use, in cardiology, telehealth’s applications are extensive, and can be used before, during, and after hospitalization. Telecardiology can be leveraged for real-time, remote diagnosis, and treatment of heart disease as well as the evaluation of congestive heart failure, cardiac arrest, and arrhythmias [72,73,78]. In psychiatry, studies have shown that telemedicine has been used more for certain diagnoses like post-traumatic stress disorder, depression, and anxiety, more than others [82]. Telepsychiatry has been found to have the potential to bridge ethnic disparities in mental health and to be beneficial among child and adolescent populations [87]. In radiology, medical doctors are trained in diagnosing and treating injuries and diseases using images acquired through various telemedicine modalities [92].

#### 5.2.5. Factors Related to Technology within the Specialty

Among specialties with lower telehealth use, providers in allergy-immunology have indicated a preference for interactive real-time video/audio technology over other modalities, due to availability of reimbursement [46,48]. Although telemedicine has been historically underleveraged in this specialty, studies show that all types of encounters are possible, including remote and synchronous encounters [48,50]. In family medicine, a majority of telehealth users have indicated preference for real-time interactive video, compared to remote monitoring, while the latter is known to be more effective for chronic disease management [64]. In gastroenterology, use of PCMHs for IBD care involves leveraging all three modalities of interactive real-time video, store-and-forward, and remote monitoring [57,62].

Among specialties with higher telehealth use, all three forms of telemedicine modalities are applicable in cardiology including interactive visits, store-and-forward for tele consultations, and remote monitoring for disease management [78,79]. In psychiatry, telemedicine is expanding beyond its original roots of interactive synchronous video into asynchronous communication [87]. In radiology, teleradiology is primarily based on store-and-forward telemedicine, i.e., the electronic capture, transmission and retrieval of images for remote viewing and interpretation [92,97].

#### 5.2.6. Factors Related to Research within the Specialty

A PubMed search of articles reporting results of telehealth-related clinical trials by specialty, over 10 years preceding the 2020 COVID-19 pandemic, revealed fewer than 20 articles in allergy-immunology and fewer than 50 in gastroenterology. On the other hand, family medicine had over 250, cardiology had over 200, psychiatry had over 600, and radiology had over 200. Overall, these results indicate specialties with lower telehealth use had a considerably lower penetration of research on telehealth outcomes, compared to specialties with higher telehealth use, with the exception of family medicine. Closer inspection revealed that over three-quarters of articles in this specialty pertained to medical homes in primary care. This suggests that, although there has been considerable research on primary care medical homes, the rate of translation of research to practice has been significantly lower, given that the concept of PCMH is still nascent in primary care practice.

#### 5.2.7. Factors Related to Culture within the Specialty

Among specialties with lower use, telehealth has historically not been considered part of mainstream practice in allergy-immunology. Although lack of reimbursement is a recognized barrier, recent literature has acknowledged that providers’ orientation to a traditional ‘gatekeeper role’ (maintaining control over treatment options), may have had a significant role to play in slowing telehealth use in within the specialty [47]. The literature revealed a similar reimbursement-driven provider culture related to telehealth use in family medicine [64,66]. Lack of reimbursement from insurers and lack of training on how to use telehealth were the most common barriers to telehealth use in family medicine. During the pandemic, the field has acknowledged that if telehealth services are to have a major impact in primary care, more family physicians will need to become experienced in using these services. Similarly, the literature discusses the general concern among providers in gastroenterology that telehealth has the potential to change the dynamics of the physician-patient relationship [59,63].

Among specialties with higher telehealth use, the provider culture associated with telehealth in cardiology can be best described as pioneering and patient-centric. Increasingly, cardiologists across the country are leveraging technology to provide virtual visits, consultations, or monitoring using a growing array of implantable or wearable devices [73,80]. Likewise, in psychiatry, a historical orientation towards maximizing patient-centered outcomes drove the early adoption of telehealth [83]. As discussed earlier, radiology was one of the earliest adopters of telemedicine, indicating an entrepreneurial and pioneering provider culture [97,98].

### 5.3. The “MICRO” Level

#### 5.3.1. Factors Related to Providers within the Specialty

Among specialties with lower telehealth use, the practices of individual providers associated with telehealth in allergy-immunology could be described as being ‘provider-centric’ practices, i.e., aligned with the traditional ‘gatekeeper’ role (described earlier), as opposed to being ‘patient-centric’ practices. During the pandemic, this field has acknowledged that allergy providers used to believe that skin tests and food challenges needed to be treated in person and that asthma could not be treated without spirometry. In a new COVID-19 era, these providers are realizing that telemedicine can be used for just about every patient and that treatment can be based purely on symptoms [47,49]. In family medicine, while general primary care provider practices could be characterized as being provider-centric, physicians engaged in primary care medical home arrangements could be described as being more patient-centric [66,69]. Similarly, in gastroenterology, while the practices of physicians engaged in traditional specialty care could be described as being more provider-centric, it could be argued that providers of IBD care in value-based specialty medical home models have embraced more patient-centric practices [54].

Among specialties with higher telehealth use, thousands of individual providers in cardiology have been reported to have embraced patient-centric care through virtual visits, teleconsultations, and remote monitoring across a variety of settings [72]. In psychiatry, patients and clinicians are reported to be largely satisfied with and engaged in telehealth use [88]. Likewise, by offering 24-h radiology services, radiology providers are known to have embraced patient-centric practices [92,98].

#### 5.3.2. Factors Related to Patients within the Specialty

As discussed earlier, among specialties with lower telehealth use, initiative by providers to educate and engage patients in telehealth use have been limited [47,58,66]. On the other hand, providers in specialties with higher telehealth use are reported to have made proactive efforts to partner with and engage patients in use of telehealth services. For example, in psychiatry, diverse patient groups have reported that they are comfortable using telepsychiatry [88]. In cardiology, remote monitoring has been found to improve confidence of older patients in managing heart failure symptoms [73,78]. Similarly, patients in radiology have benefited from improved quality and efficiency of image interpretation and lower complications [92].

## 6. Discussion

This narrative review makes an original contribution to the broader telehealth literature, by identifying a comprehensive set of *macro* (policy-level), *meso* (organizational-level), and *micro* (individual-level) factors influencing telehealth use across six medical specialties. The review is timely, because several uncertainties remain in regard to the future sustainability of telehealth services, despite the massive surge in telehealth use during the COVID-19 pandemic.

### 6.1. Summary of Findings

The review found a limited variation across the six specialties in regard to *macro*-level factors influencing telehealth use. By contrast, distinct themes were identified between specialties with lower vs. higher telehealth use, in regard to *meso*- and *micro*-level factors. For example, the review found that the historical rationale for telehealth use among specialties with lower use has been ‘improving access to care,’ which in turn, is indicative of a limited perceived importance of the potential of telehealth technology within these specialties [47,59,64]. On the other hand, the review revealed that specialties with higher telehealth use have historically leveraged telehealth services to ‘improve patient experience,’ ‘reduce costs,’ and ‘promote population health.’ Concurrently, the review revealed that, while specialties with lower telehealth use have historically received limited support from hospital and health systems, the specialties with higher use have received extensive support for telehealth use [72,82,92]. Hospital motivation to support specialties with higher use could be understood in the context of the Triple Aim framework for healthcare delivery, which translates to (1) improved patient experience, (2) lower cost, and (3) better population health. The review revealed that specialties with higher use enabled hospitals to be aligned with one or more of these aims, which in turn, helped the develop a ‘business case’ for telehealth use in those specialties. By contrast, lower using specialties did not provide hospitals with leverage to be aligned with any of the three aims.

Importantly, the review indicated that, while specialty professional societies for specialties with lower telehealth use have played a limited role in providing guidance on telehealth use, their counterparts for the specialties with higher telehealth use have played a proactive role in advocating for consistent payment policies, developing guidelines for telehealth use, educating providers on getting started with telemedicine, advocating for telehealth training in medicine residency, and developing resources for engaging patients in telehealth use [49,87]. Consistent with the leadership efforts of specialty societies, specialties with higher telehealth use like cardiology are filled with examples of provider initiatives to improve patient experience, reduce costs, and promote population health, indicating a risk-driven entrepreneurial provider culture, in contrast to a reimbursement-oriented provider culture that is aligned with the traditional ‘gatekeeper’ role [72,82,92].

By examining the influence of *micro*-, *meso*-, and *macro*-level factors on telehealth use across six specialties, this review creates the opportunity for a specialty with lower use, like allergy-immunology, to learn from a specialty with higher use, like cardiology. Although distinct themes were identified between specialties with lower and higher telehealth use across all seven factors in the *meso* layer and two factors in the micro layer, two specialty-level factors (in the *meso* layer) stand out in providing insight into actionable strategies for increasing and sustaining telehealth use at the specialty level, i.e., (1) the role of hospital organizations and (2) the role of specialty societies in influencing telehealth use within the specialty. Likewise, at the individual level (*micro* layer), the review highlighted the importance of provider-level factors, including the substantial potential of individual provider champions to influence telehealth adoption at a specialty level. Moreover, the review revealed that hospital and specialty organizations in the *meso* layer have the potential to positively impact telehealth use in the specialty (despite policy-level barriers like payment restrictions), by influencing both *macro* factors (e.g., advocating for consistent payment policies from payers) and *micro* factors (e.g., influencing provider practices to be more patient-centric and technologically savvy).

### 6.2. Implications for Widespread Sustainability of Telehealth Use

The results of this narrative review help to identify implications for ensuring the widespread sustainability of telehealth use across six medical specialties in the US. The review revealed that lack of reimbursement, lack of technology training, and a ‘gatekeeper’ mindset could all serve as barriers to telehealth adoption at the individual provider level. Hospitals and specialty societies could play an organized and proactive role in addressing each of these barriers by advocating for better payment, promulgating guidelines for telehealth use, educating providers on how to get started with telehealth, advocating for telehealth training in medical residency, and engaging patients in telehealth services. These types of organized efforts have the potential to influence providers to support more patient-centric, tech-savvy, business-oriented, and population health-focused practices. At a broader level, such initiatives have the potential to advance the Triple Aim framework of healthcare delivery, which, in turn, could create a more sustainable foundation for telehealth use on the part of providers.

Although hospital and specialty-society organizations would be reliant on consistent reimbursement for telehealth from payers, the review revealed that the former could play a proactive role in promoting telehealth use in the specialty by influencing both *macro* factors (e.g., advocating for better payment policies) and *micro* factors (e.g., influencing provider practices and culture). For example, to overcome the ‘getting started’ barrier at the provider level, specialty societies like the AAAAI could play a significant role in providing training and resources to providers within the specialty on how to effectively design and implement telehealth services in the clinic setting.

While the above can help to address telehealth sustainability issues associated with design and implementation, the sustainability of telehealth services also requires funding support beyond the pilot period, as in the case of sustained funding for gap services, urgent care services, or mandated services. For example, in cardiology, telehealth has been leveraged extensively for urgent service coverage, e.g., for percutaneous coronary intervention [99]. Similarly, telecardiology has been leveraged in Project ECHO^®^, or the Extension for Community Healthcare Outcomes, which seeks to connect specialists with primary care physicians in rural areas [100]. Specialties with lower telehealth use like allergy-immunology could learn from the cardiology experience by utilizing telehealth for mandated services, e.g., telehealth for asthma management in the correctional health setting. Additionally, given the anticipated shortage of allergy providers nationwide, the field could benefit by aligning with Project ECHO^®^ to facilitate the connection between specialists and primary physicians in rural areas. Moreover, allergy providers could be proactive in attracting support for telehealth from hospitals and payers by aligning with the Triple Aim framework to promote asthma management through remote monitoring to reduce hospitalizations, decrease costs, and promote population health. In an era of value-based reimbursement in the US, such efforts would be highly relevant to hospitals and payers seeking to expand the provision of telehealth services.

### 6.3. Limitations, Strengths, and Future Research Avenues

There are no existing studies that have empirically examined the interrelationships among all *macro*-, *meso*-, and *micro*-layer factors (examined in this review), and telehealth use at the specialty level. Most current studies on telehealth use have been cross-sectional and have examined provider use of telehealth technology within an organizational context.3, 7 Studies that have sought to examine telehealth use across specialties have focused on the association between market and structural characteristics and telehealth use [3,4,6]. A majority of the *meso*-level (specialty) factors identified in this study have not been examined in a systematic way. Correspondingly, this review provides a foundation for more holistic future research on telehealth use that takes into account a variety of policy-level, organizational-level, and individual-level factors. Future longitudinal studies may have the potential to shed light on the full impact of *meso*-level (specialty) factors examined here on telehealth use. It would be relevant to note, however, that since the findings were synthesized across six medical specialties, the potential for within-specialty variation needs to be acknowledged. For example, although psychiatry is a specialty with higher telehealth use, it has not been without its challenges of provider resistance to change [82,88]. Despite its limitations, this review is original and timely in identifying a comprehensive set of factors influencing telehealth use within a medical specialty, to provide insight into implications for ensuring widespread sustainability of telehealth use in the post-pandemic future.

## 7. Conclusions

This review paper draws upon the existing telehealth literature to develop a conceptual framework on *macro-meso-micro* factors influencing telehealth use within a medical specialty. The framework is used to guide a narrative review and synthesis of the specialty-level telehealth literature, to identify a comprehensive set of factors (barriers or facilitators) influencing telehealth use across six medical specialties in the US. The review is original in identifying a comprehensive set of policy, organizational, and individual (and interaction) factors influencing telehealth use. The results, while largely descriptive, provide insight into strategies for reducing the variation in uptake and increasing the sustainability of telehealth use across medical specialties. The review confirms that the permanent removal of *macro*-level policy barriers in the US by itself is not likely to ensure the sustainability of telehealth use at the specialty level. Instead, the review suggests that widespread and sustainable use of telehealth across medical specialties will require concerted efforts by healthcare organizations and providers to address *meso*- and *micro*-level barriers to telehealth use within the specialty. To this effect, the review highlights the crucial role that hospital and specialty-society organizations could play in creating conditions needed for successful and sustainable telehealth use at the specialty level, by concurrently addressing both the tangible barriers (e.g., reimbursement, training, workflow, design, implementation) and intangible barriers (e.g., provider attitudes, cultures) influencing telehealth use. The review is timely in that there has been substantial emphasis on telehealth adoption during COVID-19, with much benefit to public health; however, several uncertainties remain in regard to telehealth sustainability. By identifying a comprehensive set of *macro-meso-micro* factors influencing telehealth use at the specialty level, this review addresses a gap in the literature and provides a foundation for future research. Importantly, the results help to identify implications for ensuring widespread sustainability of telehealth use across medical specialties in the post-pandemic future.

## Figures and Tables

**Table 1:** Article Search Strategy on PubMed.

Search Terms	Search Results
(Allergy) AND (telehealth OR telemedicine) AND (barrier OR facilitator)	76
(Gastroenterology) AND (telehealth OR telemedicine) AND (barrier OR facilitator)	50
(Family medicine) AND (telehealth OR telemedicine) AND (barrier OR facilitator)	389
(Cardiology) AND (telehealth OR telemedicine) AND (barrier OR facilitator)	158
(Psychiatry) AND (telehealth OR telemedicine) AND (barrier OR facilitator)	593
(Radiology) AND (telehealth OR telemedicine) AND (barrier OR facilitator)	155
**Total number of records**	**1421**

**Table 2:** Key themes and insights related to *macro-meso-micro* factors influencing telehealth use.

Factor	Key Themes	Insights into Barriers and Facilitators
**The “MACRO” Level**		
**Factors related to policy and regulation**	***All six medical specialties***	
	‘National coverage and reimbursement restrictions for services offered by telemedicine.’	*Barrier*
	‘Variation in coverage and payment for telehealth services across states and private payers.’	*Barrier*
**Factors related to law and ethics**	***All six medical specialties***	
	‘State-to-state variation in policies and protocols related to provider licensure and credentialing.’	*Barrier*
	‘Concerns associated with privacy and security of data.’	*Barrier*
	‘Liability issues associated with cyber malpractice.’	*Barrier*
**Factors related to societal-level structural change**	***All six medical specialties***	
	‘Rapidly escalating costs of healthcare.’	*Barrier or Facilitator*
	‘Projected shortages in workforce.’	*Barrier or Facilitator*
	‘Demographic changes at the population level.’	*Barrier or Facilitator*
	‘Increasing preference for telehealth services among patient groups.’	*Barrier or Facilitator*
	‘Advancing technology.’	*Barrier or Facilitator*
	‘Growing use of wearable devices among patients.’	*Barrier or Facilitator*
	‘Fluctuating professional demands (e.g., need for subspecialty expertise).’	*Barrier or Facilitator*
**The “MESO” Level**		
**Factors related to perceived importance or rationale for telehealth use within the specialty**	***Specialties with lower telehealth use***	
	‘Historical rationale of increasing access to care,’ conveying limited perceived importance of telehealth use within the specialty.	*Barrier*
	***Specialties with higher telehealth use***	
	‘Historical rationale of improving patient outcomes, experience, and overall quality of care.’	*Facilitator*
	‘Historical rationale of reducing costs and/or increasing revenues’ (e.g., by decreasing inefficiencies or hospitalizations and/or by attracting patients).’	*Facilitator*
	‘Historical rationale of promoting population health.’	*Facilitator*
**Factors related to hospital/health system organization within the specialty**	***Specialties with lower telehealth use***	
	‘Hospitals or health systems have historically provided limited (low) support for telehealth use within the specialty.’	*Barrier*
	***Specialties with higher telehealth use***	
	‘Hospitals or health systems have historically provided high support for telehealth use within the specialty (to align with the Triple Aim framework).’	*Facilitator*
**Factors related to professional-society organization within the specialty**	***Specialties with lower telehealth use***	
	‘Specialty-society organization has historically provided low, reactive support for telehealth use within the specialty.’	*Barrier*
	***Specialties with higher telehealth use***	
	‘Specialty-society organization has historically provided high, proactive support for telehealth use within the specialty.’	*Facilitator*
	Specialty-society organization has historically been proactive in advancing telehealth use by influencing both *macro*-level factors (e.g., coverage or payment policies) and *micro*-level factors (e.g., provider culture and practices).’	*Facilitator*
**Factors related to treatment within the specialty**	***Specialties with lower telehealth use***	
	‘Care or treatment is based on traditional in-person encounters.’	*Barrier*
	***Specialties with higher telehealth use***	
	‘Care or treatment is designed to be holistic and patient-centered.’	*Facilitator*
**Factors related to technology within the specialty**	***Specialties with lower telehealth use***	
	‘Telemedicine technology is restricted to interactive real-time video.’	*Barrier*
	***Specialties with higher telehealth use***	
	All three telemedicine technologies (modalities) are in use, ‘interactive real-time audio/video;’ ‘store-and-forward telemedicine;’ and ‘remote patient monitoring.’	*Facilitator*
**Factors related to research within the specialty**	***Specialties with lower telehealth use***	
	‘There is limited research on outcomes related to telehealth services within the specialty.’	*Barrier*
	***Specialties with higher telehealth use***	
	‘There is extensive research on outcomes related to telehealth services within the specialty.’	*Facilitator*
**Factors related to culture within the specialty**	***Specialties with lower telehealth use***	
	‘Provider culture is driven by reimbursement’	*Barrier*
	‘Providers are resistant to telehealth use due to lack of training.’	*Barrier*
	‘Providers tend to play the traditional role of gatekeeper.’	*Barrier*
	‘Providers are concerned about changing the physician-patient relationship.’	*Barrier*
	***Specialties with higher telehealth use***	
	‘Provider culture is driven by entrepreneurialism or risk and can be characterized as pioneering and patient-centric’	*Facilitator*
**The “MICRO” Level**		
**Factors related to providers within the specialty**	***Specialties with lower telehealth use***	
	‘Telehealth practices of providers are provider-centric.’	*Barrier*
	***Specialties with higher telehealth use***	
	‘Telehealth practices of providers are patient-centric.’	*Facilitator*
**Factors related to patients within the specialty**	***Specialties with lower telehealth use***	
	‘Patients exhibit lower engagement in telehealth use.’	*Barrier*
	***Specialties with higher telehealth use***	
	‘Patients exhibit higher engagement in telehealth use.’	*Facilitator*

**Table 3:** Characteristics of 53 articles reviewed.

#	Lead Author and Year	Type of Article	Levels of Emphasis	Descriptive Summary
***Allergy-immunology***				
1	Portnoy et al., 2020 [46]	Review	Macro, Meso, Micro	Describes how the use of telemedicine, when combined with information technologies such as electronic health records, has the potential to cause a transformational change in the way care is delivered in allergy-immunology.
2	Hare et al., 2020 [47]	Specialty Workgroup Report	Macro, Meso, Micro	This work group report was developed to provide guidance to allergy-immunology clinicians as they navigate the swiftly evolving telemedicine landscape.
3	Portnoy et al., 2016 [48]	Clinical Trial	Meso, Micro	Children with asthma seen by telemedicine or in-person visits can achieve comparable degrees of asthma control.
4	Elliott et al., 2017 [49]	Position Statement	Macro, Meso, Micro	This article serves to offer policy and position statements of the use of telemedicine pertinent to the allergy and immunology subspecialty.
5	Chongmelaxme et al., 2019 [50]	Meta-Analysis	Meso, Micro	Combined telemedicine involving tele-case management or tele consultation are effective in improving asthma control and quality of life in adults.
6	Nguyen et al., 2020 [51]	Review	Micro	Providers tend to be satisfied with telemedicine if they have input into its development, there is administrative support, the technology is reliable and easy to use, as well as if there is adequate reimbursement.
7	Greiwe, 2019 [52]	Review	Micro	Telemedicine and telehealth technologies can be used to strengthen medical services and overcome many of the barriers that have previously existed by providing safe, accessible, cost-effective, and convenient healthcare at the touch of a button.
8	Shih and Portnoy, 2018 [53]	Review	Micro	Discusses the utilization of digital exam equipment, in vitro tests for diagnosis, and spirometry at the patient location; there are few clear advantages of seeing patients in-person over virtual visits.
***Gastroenterology***				
9	Click and Regueiro, 2019 [54]	Review	Macro, Meso, Micro	Explores the rationale behind initial construction of value-based IBD specialty medical homes.
10	Beard et al., 2020 [55]	Review	Macro, Meso, Micro	The future of value-based care in IBD is bright, with ample opportunities for growth.
11	Regueiro et al., 2017 [56]	Review	Meso, Micro	Describes how the IBD specialty medical home was constructed and implemented at the University of Pittsburgh Medical Center.
12	Huang et al., 2014 [57]	Meta-Analysis	Meso, Micro	This systematic review found that distance management of IBD significantly decreases clinic visit utilization.
13	Berg et al., 2020 [58]	Clinical Review	Macro, Meso, Micro	This pandemic article discusses best practice recommendations for introducing and expanding telehealth in pediatric gastroenterology.
14	Huntzinger and Bielefeldt, 2018 [59]	Program Review	Meso, Micro	This article discusses a specialty outreach program, which relied on telemedicine to reach patients with gastrointestinal and liver diseases in a large service area.
15	Allen and Kaushal, 2018 [60]	Clinical Review	Macro, Meso, Micro	Prior to 2000, a typical community GI practice comprised one to eight physicians. This article describes five new models of practice that have emerged in the past decade and have become viable choices for beginning and seasoned gastroenterologists alike.
16	Dobrusin et al., 2019 [61]	Original Research	Meso, Micro	Reports on the results of a survey of GI patients’ and physicians’ satisfaction with telehealth during the COVID-19 pandemic.
17	George and Cross, 2020 [62]	Review	Meso, Micro	The use of telehealth video conference and remote patient monitoring with web-based applications and text messaging in IBD care has been shown to ease financial burdens of chronic disease and lead to improved clinical outcomes.
18	Bilal et al., 2021 [63]	Review	Macro, Meso, Micro	Gastroenterologists need to rapidly adapt to the challenges being faced and need to make both system- and practice-based changes to the endoscopy unit and outpatient clinic practices.
***Family medicine***				
19	Moore et al., 2017 [64]	Original Research	Meso, Micro	This study found telehealth use was limited among family physicians. Lack of training and lack of reimbursement were found to be key barriers to telehealth use.
20	Powell et al., 2017 [65]	Original Research	Meso, Micro	Patients identified convenience, efficiency, communication, privacy, and comfort as domains that are potentially important to consider when assessing video visits vs. in-person encounters.
21	Krist et al., 2020 [66]	Clinical Review	Macro, Meso, Micro	Throughout the pandemic, primary care practices bore tremendous financial burden, even closing at a time when they were most needed.
22	Noel et al., 2020 [67]	Randomized Controlled Trial	Meso, Micro	Telehealth can improve transitions of care after hospital discharge by improving patient engagement and adherence to medications.
23	Phillips et al., 2015 [68]	Review	Macro, Meso, Micro	This article explores primary care health IT deployment to date, its shortcomings in support of the nation’s Triple Aim framework, and offers strategies and tactics that family medicine could pursue to improve the utility of health IT for primary care.
24	Martin et al., 2004 [69]	Original Research	Macro, Meso, Micro	System-wide changes will be needed to ensure high-quality healthcare for all Americans.
25	Rubin, 2020 [70]	Clinical Review	Macro, Meso	Discusses how the pandemic has accelerated the closure of many family practices.
26	Wakefield et al., 2016 [71]	Randomized Controlled Trial	Meso, Micro	Practices need to be selective in their use of telemonitoring with patients, limiting it to patients who have motivation for a significant change in care, such as starting insulin.
***Cardiology***				
27	Kuehn, 2016 [72]	Review	Macro, Meso, Micro	Increasingly, cardiologists across the country are leveraging technology to provide remote care.
28	Hale et al., 2016 [73]	Randomized Controlled Trial	Meso, Micro	Telehealth medication adherence technologies are a promising method to improve patient self-management.
29	Varma et al., 2020 [74]	Review	Meso, Micro	In light of the current pandemic, monitoring strategies should focus on selecting high-risk patients in need of close surveillance and using alternative remote recording devices to protect healthcare workers.
30	Schwamm et al., 2017 [75]	Position Statement	Meso, Micro	Identifies legal and regulatory barriers that impede telehealth adoption or delivery, proposes steps to overcome these barriers, and identifies areas for future research.
31	Riegel et al., 2017 [76]	Position Statement	Macro, Meso, Micro	Although there are many nuances to the relationships between self-care and outcomes, there is strong evidence that self-care is effective in achieving the goals of the treatment plan and cannot be ignored.
32	Chowdhury et al., 2020 [77]	Review	Meso, Micro	The use of adapted staffing and billing models and expanded means of remote monitoring will aid in the incorporation of telehealth into more widespread pediatric cardiology practice.
33	Dolan et al., 2020 [78]	Review	Meso, Micro	Multidisciplinary intervention resulted in decreased all-cause readmission and congestive heart failure readmission.
34	Schwamm et al., 2009 [79]	Review	Meso, Micro	Evidence-based recommendations included for various levels of care.
35	MacKinnon and Brittain, 2020 [80]	Review	Meso, Micro	MHealth is continuously developing as a result of technologic advancements and better understandings of mHealth utility.
36	Blood et al., 2020 [81]	Original Research	Meso, Micro	A navigator-led remote management strategy for optimization of guideline directed medical therapy may represent a scalable population-level strategy.
***Psychiatry***				
37	Mongelli et al., 2020 [82]	Original Research	Macro, Meso, Micro	Telepsychiatry and improvements in training of the mental health workforce are listed as useful implementations to overcome the treatment gap for patients seeking mental healthcare.
38	Yellowlees et al., 2010 [83]	Clinical Review	Meso, Micro	This article discusses guidelines ATA for the practices of tele mental health and applications for the practice of telemedicine in clinical psychiatry.
39	Shore et al., 2018 [84]	Specialty Workgroup Report	Meso, Micro	This article updates and consolidates guidance developed by The American Telemedicine Association (ATA) and The American Psychiatric Association (APA) on tele mental health services.
40	Shulman et al., 2017 [85]	Randomized Controlled Trial	Meso, Micro	A greater number of participants in the telepsychiatry group reported less subjective difficulty in keeping appointments.
41	Yellowlees et al., 2018 [86]	Randomized Controlled Trial	Meso, Micro	Describes a 5-year clinical trial comparing asynchronous telepsychiatry (ATP) with synchronous telepsychiatry (STP) consultations.
42	Yuen et al., 2015 [87]	Randomized Controlled Trial	Meso, Micro	Results suggest that prolonged exposure can be delivered via home-based telehealth with outcomes and satisfaction ratings comparable to in-person practices for certain symptoms.
43	Hubley et al., 2016 [88]	Systematic Review	Macro, Meso, Micro	A large evidence base supports telepsychiatry as a delivery method for mental health services.
44	Antonacci et al., 2008 [89]	Review	Macro, Meso, Micro	The review discusses implications for mental healthcare across settings and populations and comment on future directions and potential uses in forensic or correctional psychiatry.
45	Mahmoud and Vogt, 2019 [90]	Original Research	Macro, Meso	A comprehensive strategy to address opioid crisis, must incorporate the adoption of telepsychiatry to overcoming barriers to treatment and enhancing access to care.
46	Ramtekkar et al., 2020 [91]	Original Research	Meso, Micro	The pandemic forced a sudden shift from traditional in-person visits to alternative modalities. This paper identifies strategies and discuss considerations for long-term sustainability after the pandemic.
***Radiology***				
47	Bashshur et al., 2016 [92]	Systematic Review	Macro, Meso Micro	A consistent trend of concordance between the two modalities (teleradiology and conventional radiology) was observed in terms of diagnostic accuracy and reliability.
48	Krupinski et al., 2003 [93]	Original Research	Meso, Micro	Overall, radiologists are satisfied, although some improvements can be made.
49	Siegal et al., 2020 [94]	Position Statement	Meso, Micro	Radiology practices should be aware of the common approaches and preparations academic radiology departments have taken to reopening imaging in the post–COVID-19 disease world.
50	Johnson, 2010 [95]	Review	Macro, Meso Micro	This review aims to provide a background history to the current teleradiology services provided. It also addresses the limitations and issues involved in organizing such a service.
51	Pedrosa et al., 2020 [96]	Clinical Review	Meso, Micro	Describes the implementation of a response plan in an academic radiology department during COVID-19, challenges encountered, and tactics used to address these challenges.
52	Hryhorczuk et al., 2015 [97]	Review	Macro, Meso, Micro	Modern financial structures provide radiologists with both entrepreneurial opportunities as well as the temptation for unprofessional conduct.
53	Itri, 2015 [98]	Review	Macro, Meso, Micro	Radiologists must adapt to the changing landscape by focusing on their most important consumer: the patient.

## Data Availability

The data generated and/or analyzed in this study is included in Supplementary Materials S1.

## Supplementary Materials

The following are available online at https://www.mdpi.com/article/10.3390/ijerph18094995/s1, file S1: Appendix 1: Data Generated for Narrative Review and Synthesis.
